# The relation between passively collected data and PTSD: a systematic review and meta-analysis

**DOI:** 10.1038/s41746-025-01825-6

**Published:** 2025-07-05

**Authors:** Ningzhe Zhu, Anjali Sarawgi, Markus Bühner, Harald Baumeister, Patricia Garatva, Thomas Ehring, Yannik Terhorst

**Affiliations:** 1 Department of Psychology, LMU, Munich, Germany; 2 German Center for Mental Health (DZPG), Partner-Site Munich-Augsburg, Munich, Germany; 3 Department of Clinical Psychology and Psychotherapy, Ulm University, Ulm, Germany; 4 German Center for Mental Health (DZPG), Partner-Site Mannheim-Heidelberg-Ulm, Ulm, Germany

**Keywords:** Psychology, Human behaviour, Trauma

## Abstract

Post-traumatic stress disorder (PTSD) is a common mental disorder. This systematic review and meta-analysis examined the association between mobile sensing features and PTSD symptoms. Studies were sourced from the Database for Mobile Sensing Studies in Mental Healthcare (DAMOS), with inclusion criteria requiring correlations between mobile sensing data and PTSD symptoms assessed by validated tools. Seventeen studies encompassing 1847 participants (mean age = 38.68, 63.18% female) remained after study selection. Of 18 features across sleep, mobility, activity, and social activity, only wake after sleep onset (*r* = 0.14, 95% CI = [0.03, 0.25]) and relative amplitude of physical activity (*r* = −0.10, 95% CI = [−0.17, −0.03]) were significantly associated with PTSD symptoms. Findings were consistent across PTSD measurements, populations, demographics, and sensing durations. Although mobile sensing offers unobtrusive, objective, and ecologically valid insights into PTSD, confirmatory studies and research to optimize sensor assessment are needed before clinical practice.

## Introduction

Exposure to traumatic events is a common experience, with over 70% of people worldwide experiencing at least one in their lifetime^1,2^. Following exposure to extremely threatening or horrifying events, some develop posttraumatic stress disorder (PTSD)^3^. PTSD point prevalence is estimated at 23.81% among war survivors^4^, and the cross-national lifetime prevalence at 5.6% in the trauma-exposed general population^5^. Symptoms include distressing recollections, avoidance of thoughts, feelings, and triggers, as well as hyperarousal and changes in cognition and emotion^6^. Besides personal burden, PTSD is related to high comorbidities of other psychiatric disorders, poorer physical health, and increased medical care utilization^7–9^.

Effective PTSD treatments, such as cognitive processing therapy^10^, trauma-focused cognitive behavioral therapy^11^, and prolonged exposure therapy^12^, are well-documented. However, their initiation and success fundamentally depend on the reliable and timely assessment and identification of PTSD symptomatology^13^. Traditionally, diagnosis is based on structured clinical interviews or standardized self-reported measures^14^. The rise of wearable technology presents new opportunities for assessing and predicting mental health outcomes^15–18^. For instance, sensors in mobile phones or smartwatches may capture behaviors and symptoms in real time, potentially aiding in screening for PTSD, predicting chronicity among diagnosed individuals, and assessing behavioral features—such as avoidance and sleep disturbances—that are challenging to evaluate solely through self-report, thereby complementing standard assessments^19^. In the context of PTSD, significant progress has been made in linking various types of passively collected data to PTSD severity, with a particular focus on sleep metrics, location and mobility data, physical activity, and social activity:

Sleep disturbances, altered sleep architecture, insomnia, and nightmares are widely reported in PTSD patients^20–24^. To ensure accuracy, sleep is increasingly assessed in natural settings using polysomnography and actigraphy^25,26^. Polysomnography is an in-laboratory, overnight recording of brain waves (EEG), eye movements (EOG), muscle tone (EMG), respiration and oxygen saturation, used to quantify sleep stages, sleep continuity, and micro-arousals^27^. Actigraphy uses wrist-worn devices to track movement in the home environment, indexing rest–activity cycles, sleep onset and offset times, fragmentation, and regularity^28^. A synthesis of 20 polysomnographic studies^21^ found abnormalities in PTSD patients but reported high inconsistency and heterogeneity. Further, a meta-analysis^29^ of six actigraphy studies found no significant differences in total sleep time, wake after sleep onset, sleep efficiency, or sleep latency between those with and without PTSD. Recent research explored more advanced actigraphy-derived sleep metrics in PTSD. Some studies^30,31^ found that participants with PTSD had more fragmented sleep, while another^32^ reported reduced sleep regularity in those with more severe PTSD symptoms. However, other studies^33–36^ found no significant correlations between any sleep parameters and PTSD severity.

Location and mobility data were also found to be related to PTSD severity. PTSD symptoms, including avoidance of trauma-related places, people, or activities, and hypervigilance^37^, can be detected through GPS technology by tracking movement patterns and routines. For example, Friedmann et al. ^38^ observed a significant reduction in movement radius among PTSD patients, potentially indicating avoidance behaviors. Similarly, Ilyas et al. ^39^ reported that increased location entropy, which measures variability in time spent at various locations, correlates with reduced PTSD severity. Machine learning models have also been used to predict PTSD diagnosis with high accuracy using GPS data, such as time spent away from home and distance traveled^40,41^.

In addition to spatial considerations, PTSD has been linked to levels of physical activity. Physical activity can reduce PTSD symptoms by improving cardiorespiratory fitness and overall health^42–45^, yet many individuals with PTSD remain inactive^46^. Supporting this, a study utilizing mobile sensing data reported a negative correlation between physical activity and PTSD severity^47^. Another study found that the onset time of the most physically active period of the day is positively related to PTSD^48^. However, findings are inconsistent, as other research indicates no significant link between these variables and PTSD^30,32^. Similarly, findings on less stable and more fragmented rest-activity rhythms for PTSD patients are inconclusive^30,32,33,48^.

Social activity is also closely associated with PTSD. Robust social support networks, characterized by caring and supportive relationships, confer significant mental and physical health benefits^49,50^. Indeed, the presence of social support is a strong predictor for recovery from trauma and PTSD, playing a critical role in an individual’s ability to cope, recover, and mitigate mental health challenges^51,52^. Hence, objective sensing markers for social activity could be used to infer PTSD severity, as for instance demonstrated for the call-out counts and SMS addresses^41^.

Researchers are identifying reliable markers of PTSD or risk and protective factors impacting its course. The emerging evidence in PTSD research indicates the potential of mobile sensing for PTSD. However, there are inconsistencies on whether and how well these parameters are related to PTSD. These inconsistencies may stem from methodological heterogeneity across studies. In particular, the choice of PTSD assessment can introduce variability in how severity is defined and measured^53^. Likewise, the duration of mobile sensing windows varies widely across studies, with shorter monitoring periods potentially failing to capture stable behavioral patterns and longer protocols risking participant burden or reduced compliance^54,55^. Finally, poor reporting quality—manifested by limited disclosure of study methods, participant selection, and statistical analyses—could inflate bias in findings and heterogeneity in meta-analytic estimates^56,57^. Therefore, we conducted a systematic review of the present literature and a quantitative meta-analysis to investigate the relationship between various mobile sensing parameters and PTSD severity, and to explore potential moderating factors. Furthermore, we assessed key aspects of study quality and the likelihood of publication bias. Our primary research questions are: (1) What is the pooled correlation between different mobile sensing parameters and PTSD severity? (2) Do factors such as the method of PTSD measurement or the duration of mobile sensing influence this correlation? (3) To what extent do the studies adhere to international reporting guidelines, specifically such as the Strengthening the Reporting of Observational Studies in Epidemiology (STROBE) guidelines?

## Results

**Fig. 1:**
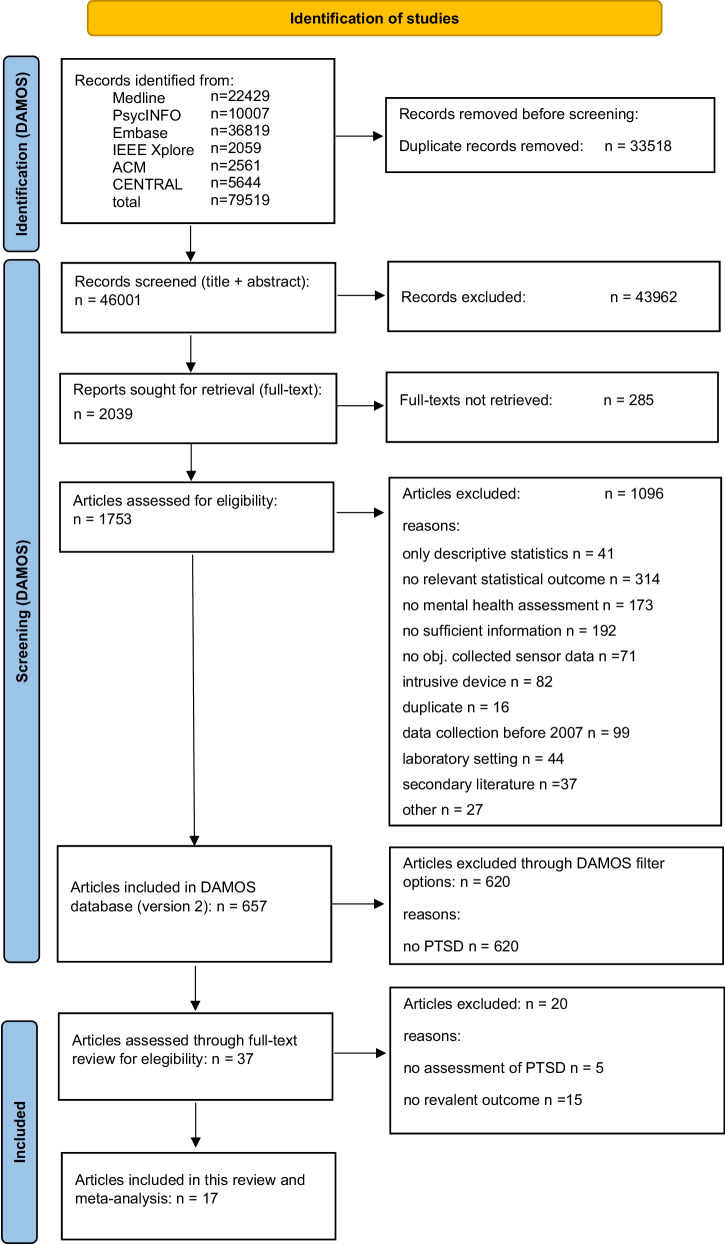
PRISMA flowchart. Thirty-seven articles were retrieved from the DAMOS database and underwent full-text review by two independent authors. The final meta-analysis included seventeen articles.

The results of the searches within the DAMOS project are presented in Fig.1. Filtering the DAMOS database (*n* = 657 articles) for PTSD resulted in 37 unique records that were related to PTSD. We excluded five studies because they did not use valid PTSD measurement tools, and another fifteen studies because they did not report effect sizes in terms of *r* and did not comply with our requests for this additional data. Consequently, 17 studies were ultimately included in our analysis. For further details, please refer to the PRISMA flowchart presented in Fig. 1.

### Study characteristics

The 17 studies included in the review encompassed 1847 participants, with sample sizes ranging from 22 to 452 (mean = 108.65, *SD* = 103.76; median = 86). The average age of participants was 38.69 years (*SD* = 14.17), and the mean percentage of female participants was 63.18% (*SD* = 31.58%). The studies included different populations: five focused on veterans (284 participants, 197 females, mean age = 50.12), two on refugees (305 participants, 154 females, mean age = 44.71), two on the general public (567 participants, 130 females, mean age = 50.48), and one each on survivors of interpersonal violence (IPV), individuals seeking treatment for substance use disorders (SUD), youths with chronic pain, child abuse victims, sexual abuse victims, ambulance paramedics, homeless youth, and individuals with histories of mental illness. For further details on the descriptive characteristics of the studies, see Table 1.

**Table 1:** Main characteristics of included studies

Reference	Country	*N*	Mean age	Gender	PTSD scale	Measurement	Device type	Device version	Period (day)	Target population	Diagnostic system
Calhoun et al. (2007)	U.S.	52	39.12	100%	DTS^a^	Self-report	Wristwatch	Mini-Mitter Actiwatch	3	Veterans	DSM-IV
Khawaja et al. (2013)	U.S.	23	52.8	86.96%	CAPS^b^ & PCL^d^	Clinical interview & Self-report	Wristwatch	Octagonal models	14	Veterans	DSM-IV
Sandahl et al. (2021)	Denmark	219	44.44	50%	HTQ^c^	Self-report	Wristwatch	Actiwatch Spectrum	14	Refugees	DSM-IV
Slightam et al. (2017)	U.S.	125	53.8	90%	CAPS^b^	Clinical interview	Wristwatch	Actiwatch-64	1	Veterans	DSM-IV
Theal et al. (2019)	Australia	40	70	0%	CAPS^b^	Clinical interview	Wrist accelerometer	wGT3X-BT monitors	14	Veterans	DSM-5
Tsanas et al. (2020)	U.K.	115	32.72	69.56%	CAPS^b^	Clinical interview	Wrist accelerometer	Geneactiv	7	General public	DSM-5
Waszczuk et al. (2020)	U.S.	452	55	11%	PCL^d^	Self-report	Waist accelerometer	ActiGraphTM WGT3X-BT	14	General public	DSM-5
Werner et al. (2016)	U.S.	51	36.1	100%	CAPS^b^	Clinical interview	Wristwatch	-	7	IPV survivors	DSM-IV
Wilkerson et al. (2021)	U.S.	22	40.4	36.70%	PCL^d^	Self-report	Wristwatch	Actiwatch Spectrum	7	SUD treatment seekers	DSM-5
Lies et al. (2021)	Australia	86	45.41	51.20%	PTSD-8^e^	Self-report	Wristwatch	Actiwatch Spectrum Pro	7	Refugees	DSM-5
Mascaro et al. (2021)	U.S.	44	33.16	28.57%	CAPS^b^ & PCL^d^	-	Wristwatch	Actiwatch 2	7	Veterans	-
Pavlova et al. (2020)	Canada	138	14.29	75%	CPSS^f^	Clinical interview	Wristwatch	Actiwatch 2	7	Youth with chronic pain	DSM-5
Friedmann et al. (2022)	Germany	160	36.21	100%	CAPS^b^	Clinical interview	Wristwatch	Move II	7	Child abuse victims	DSM-5
So et al. (2024)	U.S.	64	12.63	100%	PCL^d^	Self-report	Wristwatch	Actiwatch Spectrum Plus	7	Sexual abuse victims	DSM-5
Nguyen et al. (2023)	Australia	101	26	52%	PCL^d^	Self-report	Wristwatch	-	14	Ambulance paramedics	DSM-5
Ilyas et al. (2023)	U.S.	35	19	63%	PCL^d^	Self-report	Smartphone	-	180	Homeless youth	DSM-5
Cruz-Sanabria et al. (2023)	Italy	120	35.6	60%	TALS-SR^g^	Self-report	Wristwatch	Fitbit Flex2	7	People with mental illness histories	DSM-5

Age is reported in years; Gender is reported as the proportion of female participants. The “-” indicates not reported.

*IPV* interpersonal violence, *SUD* substance use disorder.

^a^DTS: The Davidson Trauma Scale.

^b^CAPS: The Clinician Administered PTSD Scale.

^c^HTQ: Harvard Trauma Questionnaire.

^d^PCL: PTSD Checklist for DSM-5.

^e^PTSD-8: Short Posttraumatic Stress Disorder Inventory.

^f^CPSS: Child PTSD Symptom Scale.

^g^TALS-SR: the Trauma and Loss Spectrum – Self Report.

Based on passively collected data, the studies included 18 distinct features. The number of unique studies per feature ranged from 1 to 13, with sample sizes per feature varying from 35 to 1097. A summary of these features and their definitions is provided in Table 2.

**Table 2:** Summarize of features

Feature	Definition	*k*	*N*
Sleep			
Total sleep time	The total amount of sleep time	14	1120
Wake after sleep onset	The total amount of time that a person is awake after having initially fallen asleep	12	1029
Sleep efficacy	The ratio of total sleep time to time in bed	7	570
Sleep latency	The time it takes to accomplish the transition from full wakefulness to sleep	8	541
Sleep regularity	Day-to-day consistency of sleep–wake timing	3	208
Number of awakenings	The time that a person is awake after having initially fallen asleep	4	263
Time in bed	The total amount of time in bed	2	92
Sleep entropy	Variability of activity during sleep	1	115
Mobility			
Number of clusters	The number of location clusters	1	35
Movement entropy	The variability of the time one spends across clusters	1	35
Normalized Entropy	Movement entropy normalized by the number of location clusters	1	35
Physical activity			
Interdaily stability	The degree of consistency of the rest–activity pattern from day-to-day	5	542
Intradaily variability	The degree of fragmentation of the rest–activity rhythm	5	542
M10	Activity score per hour of the most active 10-h period	4	422
M10 onset time	The beginning of M10	2	334
L5	Activity score per hour of the lest active 5-h period	3	203
Relative amplitude	The ratio between the most active 10 h and the least active 5 h	5	542
Social activity			
Wi-Fi usage	The time and type of Wi-Fi connection	1	35

The ‘*k*’’ refers to the number of effect sizes. The ‘*N*’’ refers to the total sample size that a feature includes.

### Meta-analyses results

Concerning sleep-related features, Wake After Sleep Onset (WASO) was the only feature significantly linked to PTSD symptoms, exhibiting a meta-analytically pooled correlation of *r* = 0.14 (95% CI = [0.03, 0.25], *p* = 0.014). Regarding physical activity features, relative amplitude was the only feature associated with PTSD symptoms, showing a meta-analytically pooled correlation of *r* = −0.10 (95% CI = [−0.17, −0.03], *p* = 0.016). No significant meta-analytical findings were identified for features related to mobility and social activity. Table 3 summarizes all pooled correlations, and feature-specific forest plots are available in Supplemental Fig. 1.

**Table 3:** Pooled effect sizes of included studies

Feature	*k*	*N*	*r*	95% CI	Prediction interval	I^2^	Q
Sleep							
Total sleep time	14	1120	0.02	−0.07, 0.12	−0.18, 0.23	41.70	22.30
Wake after sleep onset	12	1029	0.14*	0.03, 0.25	-0.17, 0.43	63.00	29.73**
Sleep efficacy	7	570	-0.14	−0.29, 0.03	-0.43, 0.18	57.29	14.05*
Sleep latency	8	541	0.07	−0.14, 0.27	−0.39, 0.50	69.70	23.1**
Sleep regularity	3	208	0.02	−0.35, 0.38	−0.34, 0.37	34.52	3.05
Number of awakenings	4	286	0.08	−0.30, 0.44	−0.42, 0.54	63.10	8.13*
Time in bed	2	92	-	-	-	-	-
Sleep entropy	1	115	-	-	-	-	-
Mobility							
Number of clusters	1	35	-	-	-	-	-
Movement entropy	1	35	-	-	-	-	-
Normalized Entropy	1	35	-	-	-	-	-
Physical activity							
Interdaily stability	5	542	−0.06	−0.17, 0.06	−0.18, 0.06	0.00	3.7
Intradaily variability	5	542	−0.01	−0.27, 0.25	−0.49, 0.47	78.94	18.99***
M10	4	422	−0.07	−0.24, 0.12	−0.22, 0.09	25.74	4.04
M10 onset time	2	334	-	-	-	-	-
L5	3	203	0.06	−0.03, 0.16	−0.24, 0.36	0.00	0.18
relative amplitude	5	542	−0.10*	−0.17, −0.03	−0.22, 0.02	0.00	1.32
Social activity							
Wi-Fi usage	1	35	-	-	-	-	-

The “*k*” refers to the number of effect sizes. The “*N*” refers to the total sample size that a feature includes. CI, Confidence Interval. The “-” refers to an empty value because the meta-analysis was excluded for features with fewer than three effect sizes. The “*I*^*2*^” and “*Q*” both refer to the heterogeneity of the effect sizes.

*p < 0.05. **p < 0.01. ***p < 0.001.

### Moderation analyses results

We conducted moderation analyses on the features with significant effect sizes. As shown in Table 4, there were no significant moderation effects.

**Table 4:** Results of moderation analyses

Moderator		Total sleep time			Wake after sleep onset			Sleep efficacy			Sleep latency		
Categorical moderator													
	*k*		*r*	*Q*_*b*_ (*p* value)	*k*	*r*	*Q*_*b*_ (*p* value)	*k*	*r*	*Q*_*b*_ (*p* value)	*k*	*r*	*Q*_*b*_ (*p* value)
PTSD measurement				1.46 (*p* = 0.227)			0.15 (*p* = 0.697)			1.24 (*p* = 0.265)			1.43 (*p* = 0.232)
Clinical interview	7		0.07		6	0.13		2	−0.05		3	-0.08	
Self-report	7		−0.03		6	0.17*		5	−0.17		5	0.16	
Diagnostic system				0.36 (*p* = 0.548)			1.18 (*p* = 0.278)			0.91 (*p* = 0.339)			0.33 (*p* = 0.568)
DSM-IV	5		−0.01		3	0.03		2	−0.23		3	0.11	
DSM-5	9		0.04		9	0.18*		5	−0.11		5	0.02	
Population				0.01 (*p* = 0.911)			1.18 (*p* = 0.278)			1.93 (*p* = 0.165)			0.76 (*p* = 0.526)
Veterans	5		0.04		3	0.09		1	−0.32		3	-0.06	
Non-Veterans	9		0.02		9	0.17*		6	−0.11		5	0.14	
Continuous moderator													
	*k*		b	*t* (*p* value)	*k*	b	*t* (*p* value)	*k*	b	*t* (*p* value)	*k*	b	*t* (*p* value)
Sensing duration	13		0.02	1.47 (*p* = 0.168)	12	0.02	1.47 (*p* = 0.171)	7	0.03^a^	1.79 (*p* = 0.133)	8	−0.03	−1.69 (*p* = 0.143)
Age	13		0.00	1.64 (*p* = 0.128)	12	0.00	0.69 (*p* = 0.505)	7	−0.01^a^	−2.45 (*p* = 0.059)	8	−0.01	-1.50 (*p* = 0.185)
Gender composition	13		−0.25	−1.54 (*p* = 0.149)	12	−0.34	−1.78 (*p* = 0.105)	7	−0.21^a^	−0.58 (*p* = 0.590)	8	0.47	2.16 (*p* = 0.074)

*Q*_*b*_: heterogeneity between groups. The “*k*” refers to the number of effect sizes. b: regression coefficient estimates for continuous moderators. *t*: the ratio of the estimated regression coefficient to its standard error, i.e., *t* = b/SE.

*p < 0.05.

### Research standards and small-study effects

All included studies (*k* = 17) utilized an observational study design. A comparison of these 17 published studies against the STROBE checklist revealed an overall agreement of 82.81% across all items. Only *k* = 8 (46.06%) studies reported rates of missingness for each variable of interest. Also, only *k* = 4 (23.53%) studies were preregistered, and *k* = 1 (5.88%) study presented power analysis. For all STROBE ratings, please refer to Supplemental Table 2.

Egger’s test assesses funnel-plot asymmetry by regressing the standard normal deviate of each study’s effect (i.e., the effect size divided by its standard error) on its precision (the inverse of the standard error). Under the null hypothesis of no small-study effects, the intercept of this regression should be zero. In our analysis, for all features with *k* ≥ 10 studies, the Egger intercept did not differ significantly from zero (*p* > 0.05; see Table 5), indicating no statistically detectable funnel-plot asymmetry and hence no evidence of small-study effects. For features with fewer than ten studies (*k* < 10), Egger’s test is known to have low statistical power and so may not reliably detect asymmetry. Therefore, we relied on visual inspection of the corresponding funnel plots: in most cases, the scatter of individual study estimates was evenly distributed about the pooled effect line, without a skew toward larger effects in smaller studies. For detailed funnel plots of all features, please see Supplemental Fig. 2.

**Table 5:** Egger’s test for small-study effects

Feature	Intercept	95% CI	*p*
Total sleep time	0.89	−1.35, 3.12	0.45
Wake after sleep onset	1.84	−1.98, 5.65	0.37

*CI* Confidence Interval.

## Discussion

This systematic review and meta-analysis synthesized existing research on the relationship between passively collected data and PTSD symptoms across various sensor modalities. Providing a comprehensive review of 17 studies involving 1847 participants, we focused on associations between PTSD symptoms and distinct categories of mobile sensing features, namely sleep, mobility, physical activity, and social activity. Furthermore, for features with a sufficient number of studies, meta-analyses were conducted; these revealed significant small correlations in two features related to sleep and physical activity: wake after sleep onset and relative amplitude. Additionally, we found that results were consistent across different PTSD measurements, populations, age groups, gender groups and sensing durations. Notably, no evidence of small-study effects was found, suggesting minimal publication bias and robust findings, and reporting quality was good.

Until now, many studies have investigated the link between PTSD and sleep quality metrics. However, the studies reviewed here reported heterogeneous and conflicting results, resulting in non-significant findings related to total sleep time, sleep latency, and sleep efficiency in our meta-analysis. Notably, a recent meta-analysis synthesizing actigraphy studies comparing sleep parameters between patients with clinically diagnosed PTSD and healthy controls produced different findings^58^. It found that PTSD patients exhibited lower sleep efficiency, more fragmented sleep, and an extended duration in bed. This difference from the findings of the current study may stem from the use of group differences as the measure of effect size, in contrast to our reliance on correlation metrics. Such variations might imply that, although discrepancies in these sleep parameters exist between PTSD patients and healthy individuals, a clear linear relationship between PTSD symptom severity and sleep is not evident. Considering the evidence that the severity of PTSD symptoms spans a continuum rather than a dichotomous scale^59,60^, and studies may use varying cut-off scores, a correlational approach appears more suitable. Our analysis identified wake time after sleep onset as the only sleep metric significantly correlated with PTSD symptoms. This metric reflects the duration spent awake after initially falling asleep, influenced by both frequent awakenings and difficulty returning to sleep. Evidence primarily supports the former^61–63^, highlighting the need for further research.

This review also included common GPS metrics and their relationship with PTSD symptomatology, including movement radius, location entropy, time away from home, and distance traveled^38–41^. However, meta-analyses could not be conducted on these metrics, as half of the studies incorporate these features into machine learning models, which often lack traditional effect sizes. While these models show good performance, they do not clearly define the relationship between these metrics and PTSD symptoms^40,41^. Therefore, further research is needed to explore the mechanisms underlying these associations and to determine how these metrics might be used to predict or understand PTSD symptoms more effectively. In a recent meta-analysis that pooled the correlations between passively collected GPS mobility metrics and depressive symptoms, they found small to medium correlations for many mobility features like distance traveled, normalized entropy, location variance, entropy, number of clusters, and homestay, indicating a broader application of GPS data for diagnosing depression^64^. Lots of the mobility features addressed in the review on depression have not been examined in the PTSD area. Given the high comorbidity between PTSD and depression^65^, it is essential to clearly differentiate the effects of mobility features in cases of depression alone, PTSD alone, and their comorbidity. This effort could potentially lead to more effective interventions and support systems tailored specifically to the dynamics of PTSD with and without further comorbidities.

Among five physical activity features analyzed, only relative amplitude showed a significant negative correlation with PTSD symptoms. Relative amplitude estimates the robustness of the 24-h rest-activity rhythm, by calculating the difference in activity between the most active 10-h (M10) and least active 5-h (L5) periods. Higher values indicating higher activity when awake and/or lower activity during the night^66^. This finding helps resolve discrepancies in previous studies, where some reported lower physical activity in PTSD patients while others found no difference^67^. It suggests that relative differences in activity between wake and sleep periods, rather than absolute activity levels, may be more relevant to PTSD. This aligns with research emphasizing the role of circadian rhythms in mental disorders^68^ and their potential as therapeutic targets^69^. Future studies should explore how relative amplitude affects PTSD, its predictive value, and its relevance to other stress-related conditions to refine treatment strategies.

The presence of social support plays a critical role in an individual’s ability to cope, recover, and mitigate mental health challenges^51,52^, yet PTSD often leads to detachment and emotional restriction^70,71^. Research on PTSD and sociability using wearable devices is limited. Only one study has incorporated call-out counts and SMS address counts into a machine learning model to predict PTSD^41^. Another study indirectly investigated the relationship between Wi-Fi usage and PTSD symptoms^39^. Given the critical role of social support and the limited research on mobile sensing in the context of PTSD, further investigation is needed to determine whether mobile sensing of sociability is associated with PTSD and whether it could serve as a reliable indicator in future diagnostic systems.

However, even if evidence on GPS metrics and sociability is inconclusive, overall, this review provides a meta-analytical proof-of-principle that mobile sensing metrics can capture symptoms of PTSD. Nevertheless, the identified effect sizes (WASO; *r* = 0.14; relative amplitude of physical activity: *r* = −0.10) are small according to Cohen’s criteria^72^ and future research is needed to derive optimized sensing sensors and features. For instance, studies should explore features like smartphone and app usage patterns, which have been shown to be informative for other symptoms (e.g., depression, anxiety, stress)^73–75^, but are yet lacking in the context of PTSD. Additionally, adopting a multifaceted approach that integrates various types of sensor data into a single cohort analysis may yield a more comprehensive understanding of these phenomena. To this end, mobile sensors in smartphones, rather than solely wristwatches as predominantly utilized in existing studies, are necessary: Sleep parameters, for example, can be inferred from screen usage, mobility from GPS, physical activity from movement sensors, and social activity from app usage, call logs, and messages^76,77^. Integrating data from multiple sensors enhances accuracy and could enable a more detailed digital symptom profile^78^. However, it should be noted that differences in technological factors—such as device type, firmware versions, and sensor calibrations—could influence data collection and processing and thus affect the results^79^. Moreover, so far, most studies have focused on between-person relationships, overlooking within-person variations that track PTSD severity over time. Additionally, the index events, against which PTSD symptoms are measured (e.g., mixed trauma, assault, abuse, or trauma related to job responsibilities), play a critical role in understanding PTSD^80^. Different types of traumatic events, such as interpersonal trauma (e.g., assault, abuse) and non-interpersonal trauma (e.g., accidents, natural disasters), are associated with varying risks for developing PTSD and may induce distinct symptom patterns^70,81^. However, only three of the 17 studies included in the current meta-analysis clearly reported the type of trauma involved. This limits the ability to examine whether different types of traumas relate differently to behavioral markers. For example, PTSD resulting from interpersonal trauma may be more strongly associated with social activities than PTSD resulting from non-interpersonal trauma. Therefore, it is essential for future studies to explicitly report the nature of the traumatic event. Lastly, although wearable devices can readily capture cardiovascular psychophysiology—metrics highly relevant to PTSD^82^—none of the studies in our meta-analysis employed them. Most research to date has relied on intrusive laboratory equipment, such as electrocardiograms (ECGs)^83,84^, whereas our criteria required non-intrusive sensors embedded in ubiquitous mobile devices. In fact, many consumer-grade smartwatches and rings now continuously monitor heart rate and blood oxygen level^85,86^. Future studies should therefore integrate these cardiovascular markers alongside sleep, mobility, physical activity, and social-connectivity features to develop richer, multimodal digital phenotypes.

If expanded and proven to be clinically effective, mobile sensing could transform PTSD screening and assessment by leveraging diverse passive data streams and advanced analytic frameworks. Recent studies have identified the use of sensing parameters can help improve the work of PTSD screening and assessments. For example, machine learning-based computer-aided diagnosis (CAD) systems demonstrate that video and EEG sensors alone can detect PTSD with accuracy nearing that of structured clinical interviews^87^, and fusing decomposed skin-conductance metrics with trauma-focused coping self-efficacy (CSE-T) questionnaires significantly boosts prediction of both total and cluster-level symptom change^88^. Results of the current study suggest that passive data collected by mobile sensors could serve as scalable digital markers: by flagging sleep fragmentation or circadian disruption in real time, such systems could trigger early screening prompts or clinician alerts. Moreover, the multimodal framework offers a clear path for enhancing PTSD assessments by integrating passive digital phenotypes into ensemble risk-prediction models. By combining sleep fragmentation, physical activity rhythm, mobility patterns, and social-connectivity features, future screening tools might outperform self-report alone in identifying high-risk individuals. To translate these insights into practice, researchers should (1) develop and validate ensemble classifiers that map specific sensor features onto DSM-5 symptom clusters (e.g., using sleep metrics to index hyperarousal and mobility metrics to index avoidance), (2) compare their performance against standalone questionnaires and structured interviews, and (3) assess incremental utility by quantifying how much diagnostic accuracy improves when adding each passive data stream. Critically, moving beyond between-person correlations will require within-subject, longitudinal designs: repeated, time-linked measures of both passive sensing and symptom severity can establish whether fluctuations in digital markers reliably precede—or merely reflect—changes in PTSD symptoms. Such efforts will clarify temporal dynamics, enhance causal inference, and could enable personalized just-in-time-adaptive treatment. Of course, confirmatory studies are needed, and acceptance, ethical and privacy challenges must be overcome before such mobile sensing augmented assessments can be recommended for clinical practice^18,89–92^.

Lastly, we would like to highlight a few important points, that warrant attention when interpreting the present findings. First, this meta-analysis relies on studies reporting total PTSD severity scores. They treated PTSD as a unidimensional construct, summing across all symptoms and thereby obscuring the disorder’s heterogeneity. In fact, under DSM-5 criteria, there are an estimated 79,794 combinations of symptom profiles^93^. Summed-score approaches assume that all symptoms covary linearly with any given predictor (e.g., a sensor metric), but if a passive-sensing feature is specifically related to one cluster (e.g., sleep fragmentation and intrusion symptoms) and unrelated to others (e.g., avoidance), correlating it with a total severity score will attenuate or even erase that association. Future studies should move beyond total severity and leveraging the hierarchical, dimensional structure of psychopathology like HiTOP^94^. We can therefore detect and interpret the nuanced relationships between passive mobile-sensing features and specific PTSD symptom dimensions. Second, the correlation-based meta-analyses used in this study exhibited relatively low power when estimating small amounts of effect sizes (ranging from 3 to 14). This limitation constrained our ability to draw definitive conclusions. In the future, as more studies and datasets become available, research in this area could benefit from combining individual participant data with aggregated study-level data in meta-analyses of correlational studies^95^. Also, the present meta-analysis only tested linear relationships between variables. Since previous meta-analyses yielded different findings when examining group differences^58^, future research should explore nonlinear models that predict PTSD symptoms based on mobile sensing features. Third, the sample sizes in the included studies (ranging from 22 to 452, with a median of 86) are generally too small to reliably detect assumable small to moderate correlations with sufficient power, indicating a need for larger studies (e.g., guided in sample size planning by the here identified meta-analytical correlations and their confidence intervals). Fourth, most studies were conducted in high-income Western countries, which may limit the generalizability of the findings. Future research should expand to include low- and middle-income countries and regions in Eastern countries to explore potential financial and cultural influences on the results. Furthermore, reporting standards in this area need to be improved. The reporting in studies addressed STROBE items in most cases (82.81%), but deficits were found in addressing missing data and power analyses. Standardized international protocols for feature calculation, reporting, and handling missing data are crucial for advancing this research field.

This study represents the first meta-analysis providing robust evidence that mobile sensing features related to sleep and physical activity are correlated with PTSD symptoms. Besides, there exists sparse evidence in individual studies that mobility and social activity are also associated with PTSD symptoms. As a proof-of-principle for mobile sensing in the context of PTSD, this lays the foundation for future research in the field. To comprehensively delineate the potential of mobile sensing in individuals with PTSD, further high-quality data in larger studies are required covering the so far studied four aspects (sleep metrics, location and mobility, physical activity, social activity), additional sensor modalities (e.g., app usage, smartphone usage, and language usage), symptom-specific analysis, and a ideally a within-person perspective in addition to the here identified between-person studies. With this effort, and if reliable objective mobile sensing markers are proven to be effective in confirmatory studies, mobile sensing may in the future enable real-time, passive monitoring of PTSD symptoms for individuals who have experienced trauma and facilitate timely interventions and personalized treatments.

## Methods

The methodology for this systematic review adheres to the preferred reporting items for systematic reviews and meta-analyses (PRISMA^96^) guidelines (see Supplemental Table 1 for details) and preregistered at the open science framework (https://osf.io/w2urq). This systematic review and meta-analysis is part of the Database for Mobile Sensing Studies in Mental Healthcare project (DAMOS^97^, registered at https://osf.io/5ukt9/). The DAMOS project aims to establish a comprehensive and sustainably maintained open science database for studies on mobile sensing dedicated to mental healthcare^97^.

### Identification and selection of studies

A systematic literature search was conducted on 20 February 2024 as part of the DAMOS project^97^, utilizing the bibliographic databases Medline, Embase, PsycINFO, CENTRAL, IEEE Xplore, and ACM. The search string focused on key concepts related to mobile sensing, used sensory, and a broad range of mental disorders including amongst others PTSD. The Medline search string can be found in the DAMOS project repository^97^.

To be included in the DAMOS database, studies had to meet the following criteria: (1) Involve a human population across the lifespan, (2) Data collection from 2007 or later, (3) Quantitative data collection using at least one smart sensor, (4) Use of smart sensors that are embedded in a non-intrusive wearable device suitable for everyday use, (5) Statistical outcomes reporting mental disorders in relation to sensor data, (6) Assessment of mental disorders using a validated clinical instrument, and (7) Original empirical research.

For this study, the DAMOS database (version 2)^97^ was filtered to specifically retrieve articles that included an assessment of PTSD, as our search was aimed at identifying studies that examined associations between passively collected wearable device data (e.g., smartwatches) and PTSD symptoms.

### Eligibility criteria

The inclusion criteria for this review were as follows: studies had to (1) focus on human participants of any age, (2) collect data in or after 2007, (3) gather quantitative data using at least one smart sensor embedded in an unobtrusive wearable device available and suitable in daily life, (4) assess PTSD either by self-report or by structured clinical interview, and (5) report empirical analyses on collected sensor data and PTSD severity (e.g., correlation and regression).

### Data extraction

Data extraction was performed independently by two authors (N.Z. and A.S.) and included effect sizes and study characteristics. Any disagreements were resolved through discussion or by consulting a third author (Y.T.). To estimate effect sizes, we utilized the zero-order correlation (*r*). We converted regression coefficients (β) to *r*, adhering to the guidelines delineated by Peterson & Brown^98^. Regarding studies that reported statistics other than *r*, we contacted the corresponding authors for the missing information through repeated email containing a study description and extraction template for the needed information.

For study characteristics, the authors’ names, publication year, measures of PTSD (e.g., Posttraumatic Stress Disorder Checklist for DSM-5 [PCL-5]), sensor features (e.g., total sleep time), sensing duration and sample characteristics (e.g., age, gender, and country) were coded.

### Statistical analysis

We conducted meta-analyses on all features that were presented in more than three studies. All of our analyses were performed in R Version 4.2.3^99^ utilizing the “metafor” package developed by Viechtbauer^100^. To obtain a more generalized outcome, we utilized a random-effects model that considers the possible variation among the included studies. Separate analyses were conducted for each feature (e.g., total sleep time, sleep latency, and wake after sleep onset), with pooled results reported individually. We also performed subgroup analyses to investigate categorical moderators (i.e., PTSD measurement and population) and meta-regression to explore continuous moderators (i.e., age, Gender composition and sensing duration). Heterogeneity was assessed using *I*² and *Q* statistics.

### Assessment of research standards and small-study effects

As a general criterion for adherence to international reporting guidelines, we assessed the reference and adherence to the STROBE^101^ guidelines of the included observational studies for an approximation of reporting and study quality. The STROBE checklist was rated for each study by two independent researchers (N.Z. and A.S.). Disagreements were resolved in discussion.

We assessed small-study effects using two different approaches: visual examination of the funnel plot symmetry^102^ and the Egger’s regression test^103^. For the funnel plot, we plotted the observed effect sizes of individual studies on the x-axis against their standard errors (inverted on the y-axis to represent precision). In the absence of bias, the plot is expected to form a symmetrical, inverted funnel shape. For Egger’s test, we fitted a linear regression model where the standardized effect sizes (effect sizes divided by their standard errors) were regressed against the precision (the inverse of the standard errors). This test evaluates whether the regression intercept differs significantly from zero, which would suggest funnel plot asymmetry and possible small-study effects. For a more detailed introduction to these methods, see ref. ^104^.

## Supplementary information

Supplementary information

## Data Availability

The dataset extracted and used in the final analysis are online at https://osf.io/am3py/files/osfstorage.

## Code availability

The code used to analyze the data and reproduce results can be found at https://osf.io/am3py/files/osfstorage.
