# Supplementary material for: The relation between passively collected data and PTSD: a systematic review and meta-analysis
Source: NPJ Digit Med. 2025 Jul 5;8:413. doi: 10.1038/s41746-025-01825-6 (PMC12228780; doi:10.1038/s41746-025-01825-6)

Checklist of supplemental figures and tables.

Supplemental Table 1.	PRISMA 2020 Checklist.
Supplemental Figure 1.	Forest plots of all feature groups.
Supplemental Table 2.	STROBE adherence checklist.
Supplemental Figure 2.	Funnel plots of all feature groups.

Supplemental Table 1. PRISMA 2020 Checklist

Abstract

Section and Topic	Item #	Checklist item	Reported (Yes/No)
**TITLE**			
Title	1	Identify the report as a systematic review.	Yes
**BACKGROUND**			
Objectives	2	Provide an explicit statement of the main objective(s) or question(s) the review addresses.	Yes
**METHODS**			
Eligibility criteria	3	Specify the inclusion and exclusion criteria for the review.	Yes
Information sources	4	Specify the information sources (e.g. databases, registers) used to identify studies and the date when each was last searched.	Yes
Risk of bias	5	Specify the methods used to assess risk of bias in the included studies.	Quality assessment via STROBE.
Synthesis of results	6	Specify the methods used to present and synthesise results.	Yes
**RESULTS**			
Included studies	7	Give the total number of included studies and participants and summarise relevant characteristics of studies.	Yes
Synthesis of results	8	Present results for main outcomes, preferably indicating the number of included studies and participants for each. If meta-analysis was done, report the summary estimate and confidence/credible interval. If comparing groups, indicate the direction of the effect (i.e. which group is favoured).	Yes
**DISCUSSION**			
Limitations of evidence	9	Provide a brief summary of the limitations of the evidence included in the review (e.g. study risk of bias, inconsistency and imprecision).	Yes
Interpretation	10	Provide a general interpretation of the results and important implications.	Yes
**OTHER**			
Funding	11	Specify the primary source of funding for the review.	Yes
Registration	12	Provide the register name and registration number.	Yes

From: Page MJ, McKenzie JE, Bossuyt PM, Boutron I, Hoffmann TC, Mulrow CD, et al. The PRISMA 2020 statement: an updated guideline for reporting systematic reviews. BMJ 2021;372:n71. doi: 10.1136/bmj.n71

Paper

Section and Topic	Item #	Checklist item	Location where item is reported
**TITLE**			
Title	1	Identify the report as a systematic review.	1
**ABSTRACT**			
Abstract	2	See the PRISMA 2020 for Abstracts checklist.	2-3
**INTRODUCTION**			
Rationale	3	Describe the rationale for the review in the context of existing knowledge.	4
Objectives	4	Provide an explicit statement of the objective(s) or question(s) the review addresses.	7
**METHODS**			
Eligibility criteria	5	Specify the inclusion and exclusion criteria for the review and how studies were grouped for the syntheses.	8-9
Information sources	6	Specify all databases, registers, websites, organisations, reference lists and other sources searched or consulted to identify studies. Specify the date when each source was last searched or consulted.	7
Search strategy	7	Present the full search strategies for all databases, registers and websites, including any filters and limits used.	7-8
Selection process	8	Specify the methods used to decide whether a study met the inclusion criteria of the review, including how many reviewers screened each record and each report retrieved, whether they worked independently, and if applicable, details of automation tools used in the process.	9
Data collection process	9	Specify the methods used to collect data from reports, including how many reviewers collected data from each report, whether they worked independently, any processes for obtaining or confirming data from study investigators, and if applicable, details of automation tools used in the process.	9
Data items	10a	List and define all outcomes for which data were sought. Specify whether all results that were compatible with each outcome domain in each study were sought (e.g. for all measures, time points, analyses), and if not, the methods used to decide which results to collect.	9
	10b	List and define all other variables for which data were sought (e.g. participant and intervention characteristics, funding sources). Describe any assumptions made about any missing or unclear information.	9

Study risk of bias assessment	11	Specify the methods used to assess risk of bias in the included studies, including details of the tool(s) used, how many reviewers assessed each study and whether they worked independently, and if applicable, details of automation tools used in the process.	10-11
Effect measures	12	Specify for each outcome the effect measure(s) (e.g. risk ratio, mean difference) used in the synthesis or presentation of results.	9
Synthesis methods	13a	Describe the processes used to decide which studies were eligible for each synthesis (e.g. tabulating the study intervention characteristics and comparing against the planned groups for each synthesis (item #5)).	9
	13b	Describe any methods required to prepare the data for presentation or synthesis, such as handling of missing summary statistics, or data conversions.	9
	13c	Describe any methods used to tabulate or visually display results of individual studies and syntheses.	9
	13d	Describe any methods used to synthesize results and provide a rationale for the choice(s). If meta-analysis was performed, describe the model(s), method(s) to identify the presence and extent of statistical heterogeneity, and software package(s) used.	9
	13e	Describe any methods used to explore possible causes of heterogeneity among study results (e.g. subgroup analysis, meta-regression).	10
	13f	Describe any sensitivity analyses conducted to assess robustness of the synthesized results.	10-11
Reporting bias assessment	14	Describe any methods used to assess risk of bias due to missing results in a synthesis (arising from reporting biases).	10-11
Certainty assessment	15	Describe any methods used to assess certainty (or confidence) in the body of evidence for an outcome.	10-11
**RESULTS**			
Study selection	16a	Describe the results of the search and selection process, from the number of records identified in the search to the number of studies included in the review, ideally using a flow diagram.	11, Fig. 1
	16b	Cite studies that might appear to meet the inclusion criteria, but which were excluded, and explain why they were excluded.	11
Study characteristics	17	Cite each included study and present its characteristics.	11-12, Table 1
Risk of bias in studies	18	Present assessments of risk of bias for each included study.	13
Results of individual studies	19	For all outcomes, present, for each study: (a) summary statistics for each group (where appropriate) and (b) an effect estimate and its precision (e.g. confidence/credible interval), ideally using structured tables or plots.	12-13, Supplement 2

Results of syntheses	20a	For each synthesis, briefly summarise the characteristics and risk of bias among contributing studies.	13
	20b	Present results of all statistical syntheses conducted. If meta-analysis was done, present for each the summary estimate and its precision (e.g. confidence/credible interval) and measures of statistical heterogeneity. If comparing groups, describe the direction of the effect.	12,<br>Table 3
	20c	Present results of all investigations of possible causes of heterogeneity among study results.	13,<br>Table 4
	20d	Present results of all sensitivity analyses conducted to assess the robustness of the synthesized results.	NA
Reporting biases	21	Present assessments of risk of bias due to missing results (arising from reporting biases) for each synthesis assessed.	13
Certainty of evidence	22	Present assessments of certainty (or confidence) in the body of evidence for each outcome assessed.	12
**DISCUSSION**			
Discussion	23a	Provide a general interpretation of the results in the context of other evidence.	14
	23b	Discuss any limitations of the evidence included in the review.	17
	23c	Discuss any limitations of the review processes used.	17
	23d	Discuss implications of the results for practice, policy, and future research.	17
**OTHER INFORMATION**			
Registration and protocol	24a	Provide registration information for the review, including register name and registration number, or state that the review was not registered.	10
	24b	Indicate where the review protocol can be accessed, or state that a protocol was not prepared.	10
	24c	Describe and explain any amendments to information provided at registration or in the protocol.	NA
Support	25	Describe sources of financial or non-financial support for the review, and the role of the funders or sponsors in the review.	19
Competing interests	26	Declare any competing interests of review authors.	19
Availability of data, code and other materials	27	Report which of the following are publicly available and where they can be found: template data collection forms; data extracted from included studies; data used for all analyses; analytic code; any other materials used in the review.	10

Supplemental Figure 1. Forest plots of all feature groups.

A. Total sleep time

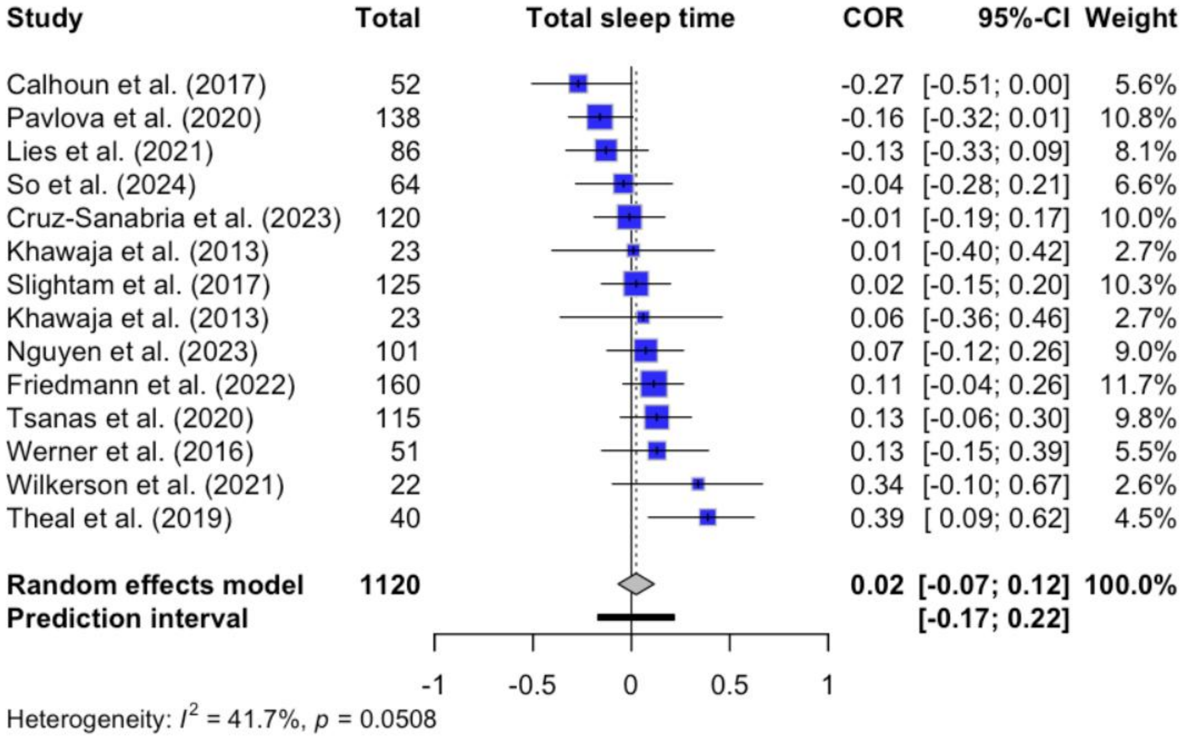

B. Wake after sleep one set (WASO)

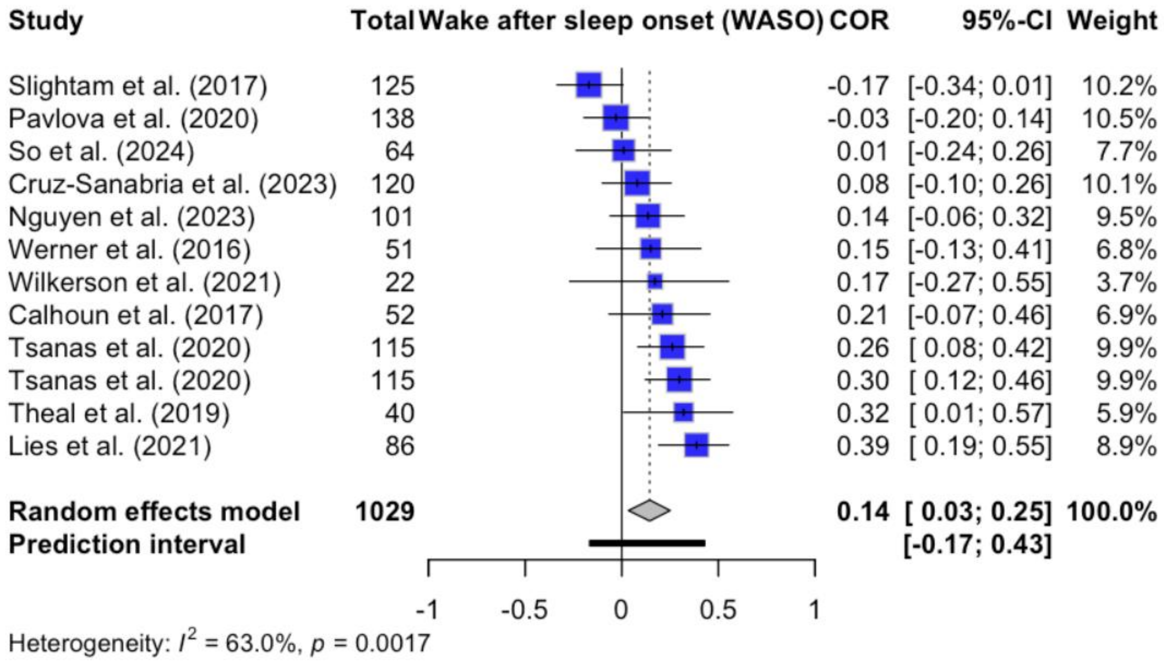

C. Sleep efficacy

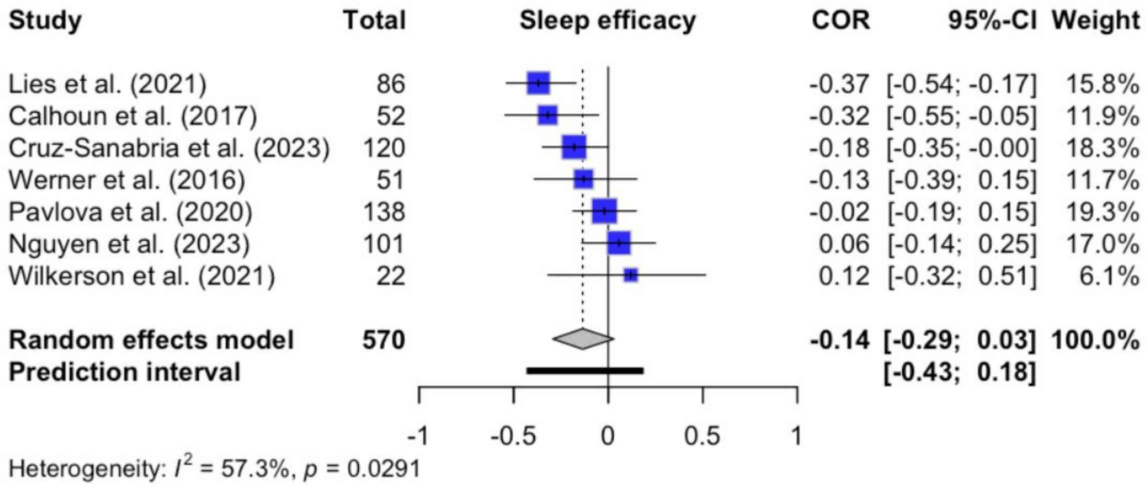

D. Sleep latency

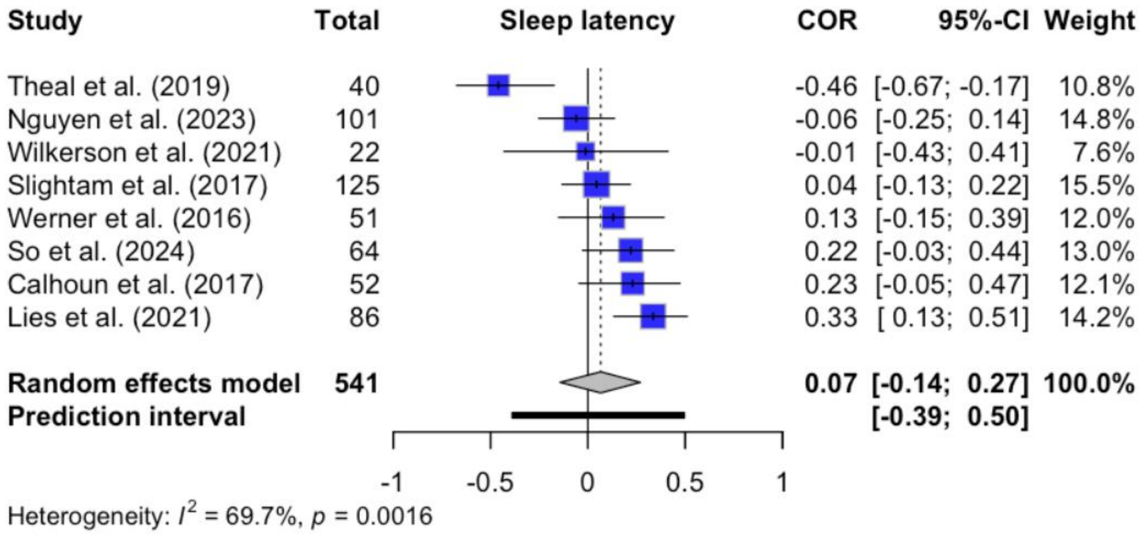

E. Sleep regularity

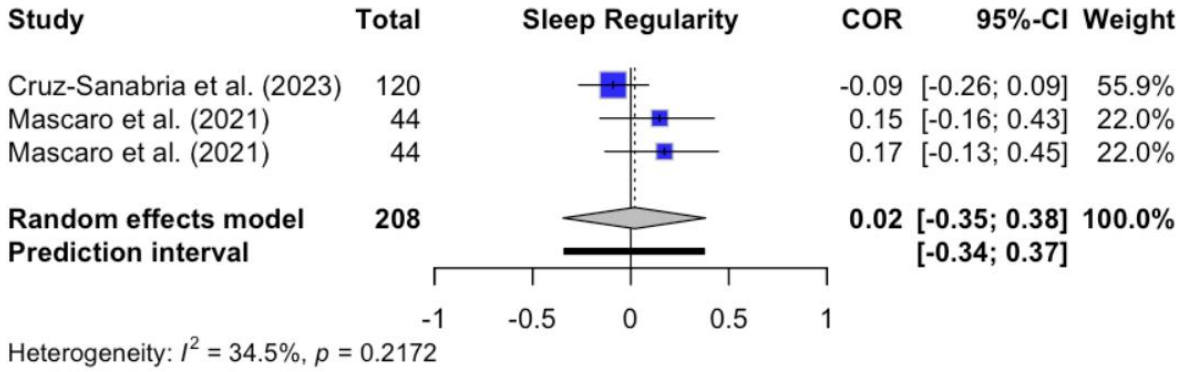

F. Number of awakenings

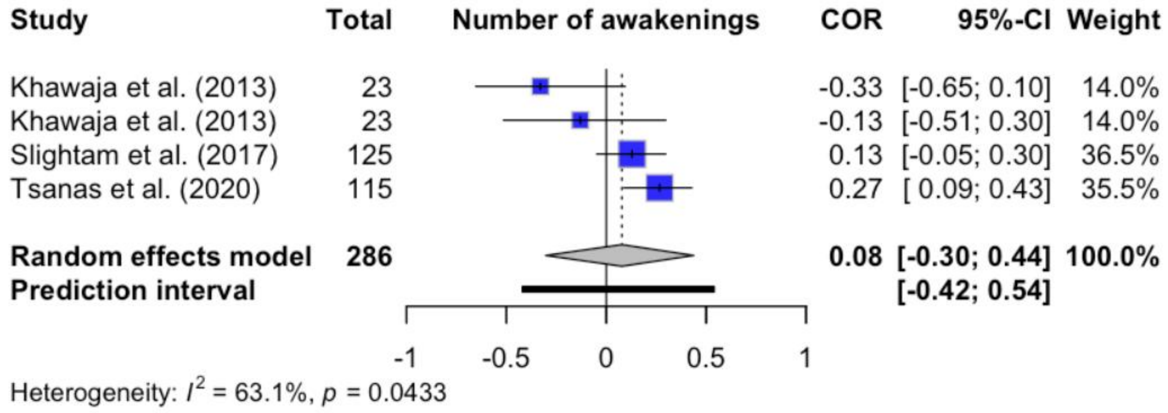

G. Interdaily stability

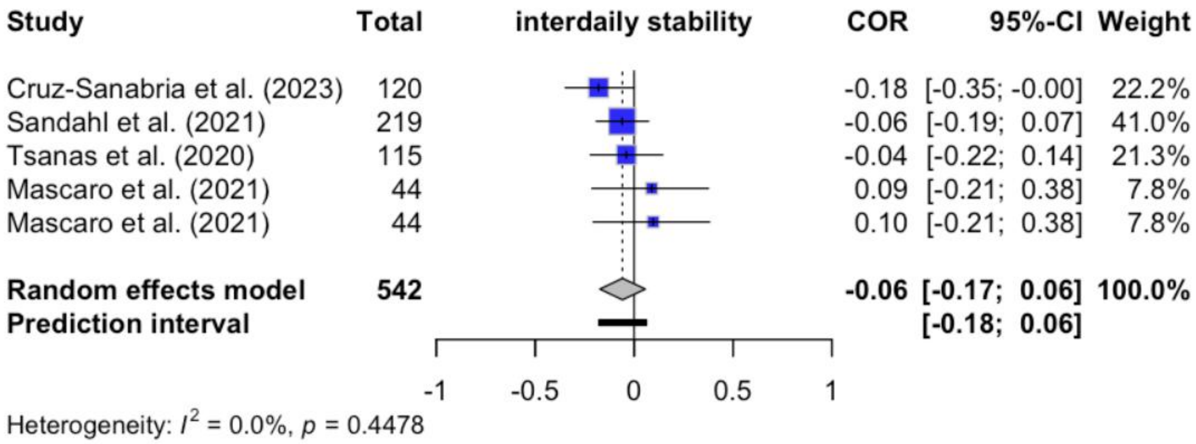

H. Intradaily variability

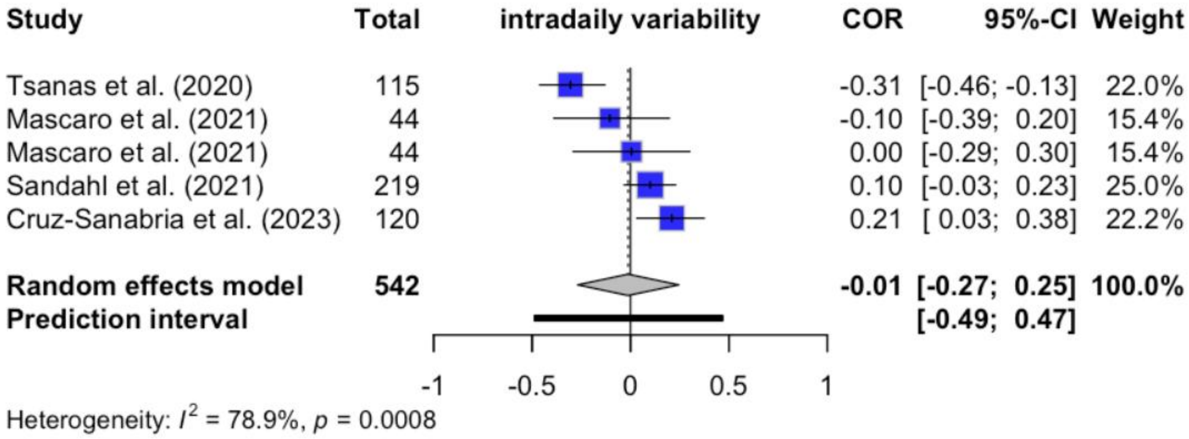

I. Most active 10 h period (M10)

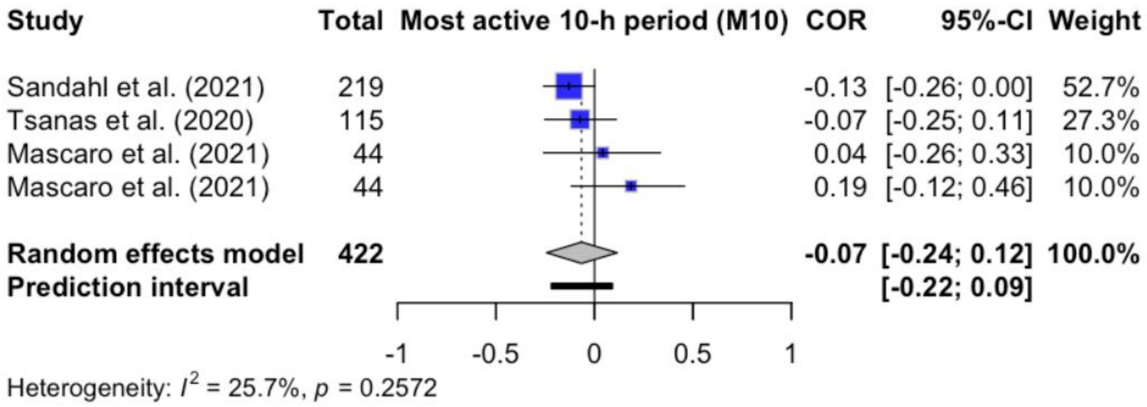

J. Least active 5 hour period (L5)

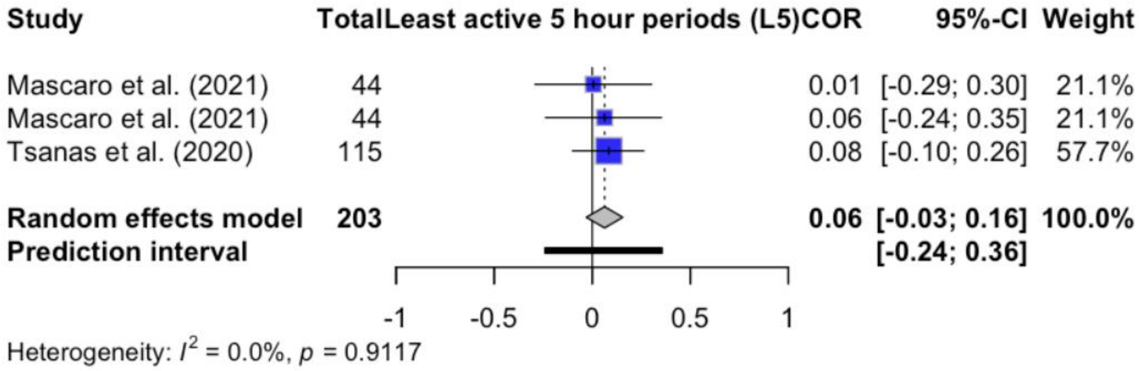

K. Relative amplitude

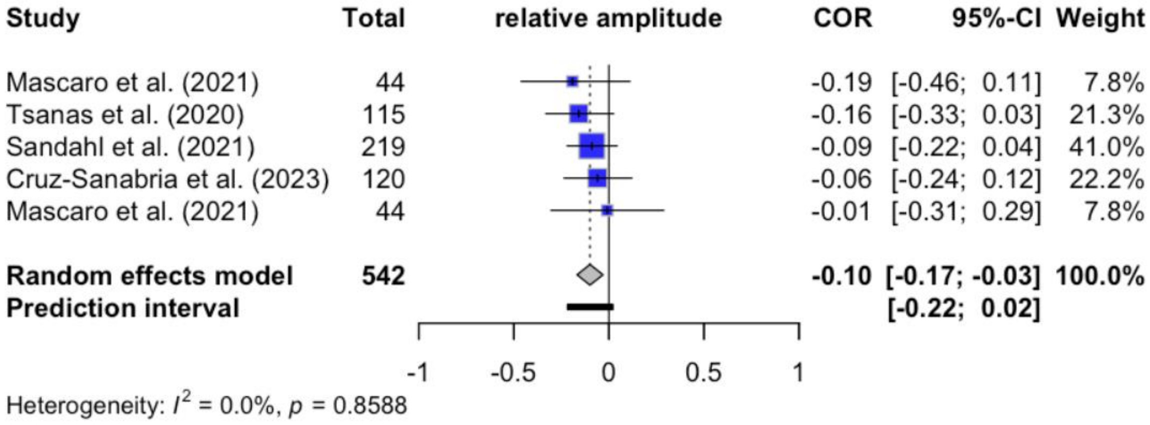

Supplemental Table 2. STROBE adherence checklist.

	Title and abstract		Introduction		Methods														
	design	abstract	Background/rationale	Objectives	Study design	Setting	Participants					Variables	Data sources/ measurement	Bias	Study size	Quantitative variables	Statistical methods		
Item No	1		2	3	4	5	6					7	8*	9	10	11			
Recommendation	(*a*) Indicate the study's design with a commonly used term in the title or the abstract	(*b*) Provide in the abstract an informative and balanced summary of what was done and what was found	Explain the scientific background and rationale for the investigation being reported	State specific objectives, including any prespecified hypotheses	Present key elements of study design early in the paper	Describe the setting, locations, and relevant dates, including periods of recruitment, exposure, follow-up, and data collection	(*a*) *Cohort study* —Give the eligibility criteria, and the sources and methods of selection of participants. Describe methods of follow-up	*Case-control study* —Give the eligibility criteria, and the sources and methods of case ascertainment and control selection. Give the rationale for the choice of cases and controls	*Cross-sectional study* —Give the eligibility criteria, and the sources and methods of selection of participants	(*b*) *Cohort study* —For matched studies, give matching criteria and number of exposed and unexposed	*Case-control study* —For matched studies, give matching criteria and the number of controls per case	Clearly define all outcomes, exposures, predictors, potential confounders, and effect modifiers. Give diagnostic criteria, if applicable	For each variable of interest, give sources of data and details of methods of assessment (measurement). Describe comparability of assessment methods if there is more than one group	Describe any efforts to address potential sources of bias	Explain how the study size was arrived at	Explain how quantitative variables were handled in the analyses. If applicable, describe which groupings were chosen and why	(*a*) Describe all statistical methods, including those used to control for confounding	(*b*) Describe any methods used to examine subgroups and interactions	(*c*) Explain how missing data were addressed
Studie																			
Calhoun et al., 2007	0	1	1	1	1	0	na	1	na	na	na	1	1	1	0	1	0	0	0
Khawaja et al., 2013	0	1	1	1	1	0	na	na	1	na	na	1	1	0	0	1	0	0	0
Sandahl et al., 2021	0	1	1	1	1	1	na	na	1	na	na	1	1	1	1	1	1	1	1
Slightam et al., 2017	0	1	1	1	1	0	na	1	na	na	na	1	1	1	1	1	1	1	1
Theal et al., 2019	1	1	1	1	1	1	na	na	1	na	na	1	1	1	0	1	1	1	0
Tsanas et al., 2020	0	1	1	1	1	0	na	na	1	na	na	1	1	1	0	1	1	1	1
Waszczuk et al., 2020	1	1	1	1	1	0	na	na	1	na	na	1	1	1	0	1	1	1	0
Werner et al., 2016	0	1	1	1	1	0	1	na	na	na	na	1	1	1	1	1	1	1	1
Wilkerson et al., 2021	0	1	1	1	1	1	1	na	na	na	na	1	1	0	0	1	1	0	0
Lies et al., 2021	1	1	1	1	1	1	na	na	1	na	na	1	1	1	1	1	1	0	1
Mascaro et al., 2021	0	1	1	1	1	0	na	na	1	na	na	1	1	1	0	1	1	1	1
Pavlova et al., 2020	1	1	1	1	1	1	1	na	na	na	na	1	1	1	0	0	1	0	1
So et al., 2024	0	1	1	1	1	0	na	na	1	na	na	1	1	0	0	1	1	1	1
Nguyen et al., 2023	1	1	1	1	1	1	1	na	na	na	na	1	1	1	1	1	1	1	1
Ilyas et al., 2023	1	1	1	1	1	1	na	na	1	na	na	1	1	1	1	1	1	1	1
Cruz-Sanabria et al., 2023	0	1	1	1	1	1	na	na	1	na	na	1	1	1	0	1	1	1	0
Friedmann et al., 2022	0	1	1	1	1	1	na	na	1	na	na	1	1	1	1	1	1	1	1
adherence	6	17	17	17	17	9	4	2	11	0	0	17	17	14	7	16	15	12	11
elegible	17	17	17	17	17	17	4	2	11	0	0	17	17	17	17	17	17	17	17
percent	35.29%	100.00%	100.00%	100.00%	100.00%	52.94%	100.00%	100.00%	100.00%	#DIV/0!	#DIV/0!	100.00%	100.00%	82.35%	41.18%	94.12%	88.24%	70.59%	64.71%
0.82806 AverageInSection		0.676471		1		0.784314													
	Title and abstract		Introduction		Methods														

				results													discussion				other
				Participants			Descriptive data			Outcome data			Main results			Other analyses	Key results	Limitations	Interpretation	Generalisability	Funding
12				13*			14*			15*			16			17	18	19	20	21	22
(*d*) *Cohort study* —If applicable, explain how loss to follow-up was addressed	*Case-control study* —If applicable, explain how matching of cases and controls was addressed	*Cross-sectional study* —If applicable, describe analytical methods taking account of sampling strategy	(*e*) Describe any sensitivity analyses	(a) Report numbers of individuals at each stage of study—eg numbers potentially eligible, examined for eligibility, confirmed eligible, included in the study, completing follow-up, and analysed	(b) Give reasons for non-participation at each stage	(c) Consider use of a flow diagram	(a) Give characteristics of study participants (eg demographic, clinical, social) and information on exposures and potential confounders	(b) Indicate number of participants with missing data for each variable of interest	(c) *Cohort study* —Summarise follow-up time (eg, average and total amount)	*Cohort study* —Report numbers of outcome events or summary measures over time	*Case-control study*— Report numbers in each exposure category, or summary measures of exposure	*Cross-sectional study*— Report numbers of outcome events or summary measures	(*a*) Give unadjusted estimates and, if applicable, confounder-adjusted estimates and their precision (eg, 95% confidence interval). Make clear which confounders were adjusted for and why they were included	(*b*) Report category boundaries when continuous variables were categorized	(*c*) If relevant, consider translating estimates of relative risk into absolute risk for a meaningful time period	Report other analyses done—eg analyses of subgroups and interactions, and sensitivity analyses	Summarise key results with reference to study objectives	Discuss limitations of the study, taking into account sources of potential bias or imprecision. Discuss both direction and magnitude of any potential bias	Give a cautious overall interpretation of results considering objectives, limitations, multiplicity of analyses, results from similar studies, and other relevant evidence	Discuss the generalisability (external validity) of the study results	Give the source of funding and the role of the funders for the present study and, if applicable, for the original study on which the present article is based
na	0	na	0	1	1	0	1	0	na	na	1	na	1	na	na	0	1	1	1	1	0
na	na	0	0	1	1	0	1	0	1	1	na	na	0	1	na	0	1	1	1	1	1
na	na	0	0	0	0	0	1	0	na	na	1	na	1	na	na	0	1	1	1	1	1
na	1	na	0	1	1	0	1	0	na	na	1	na	1	0	na	0	1	1	1	1	1
na	na	0	1	0	0	0	1	0	na	na	na	1	0	1	na	0	1	1	1	1	1
na	na	0	0	0	0	0	1	1	na	na	na	1	0	na	na	0	1	1	1	1	1
na	na	0	0	0	0	0	1	0	na	na	na	1	1	na	na	0	1	1	1	1	1
na	na	na	1	1	1	0	1	1	1	1	na	na	1	1	na	1	1	1	1	1	1
1	na	na	1	1	0	0	1	0	1	1	na	na	1	na	na	1	1	1	1	1	1
na	na	1	1	1	0	0	1	1	na	na	na	1	1	1	na	1	1	1	1	1	0
na	na	0	0	0	0	0	1	1	na	na	na	1	0	na	na	0	1	1	1	0	1
1	na	na	1	1	0	1	1	1	1	1	na	na	0	1	na	1	1	1	1	1	1
na	na	0	0	1	1	0	1	1	na	na	na	1	1	1	na	1	1	1	1	1	1
1	na	na	1	1	1	1	1	1	1	1	na	na	1	1	na	1	1	1	1	1	1
na	na	0	1	1	1	1	1	0	na	na	na	1	1	0	na	1	1	1	1	1	1
na	na	0	0	0	0	0	1	0	na	na	na	1	1	1	na	0	1	1	1	1	1
na	na	0	1	1	1	0	1	1	1	1	na	na	1	1	na	1	1	1	1	1	1
3	1	1	8	11	8	3	17	8	6	6	3	8	12	9	0	8	17	17	17	16	15
3	2	11	17	17	17	17	17	17	6	6	3	8	17	11	0	17	17	17	17	17	17
100.00%	50.00%	9.09%	47.06%	64.71%	47.06%	17.65%	100.00%	47.06%	100.00%	100.00%	100.00%	100.00%	70.59%	81.82%	#DIV/0!	47.06%	100.00%	100.00%	100.00%	94.12%	88.24%
					0.639929													0.985294			88.24%
				results													discussion				other

custom				
Explicit study design	STROBE	Registration	Protocol	Power
custom	custom	custom	custom	Custom
Explicit study design	STROBE reference?	registration ?	protocol?	Power analysis present in paper (a-priori)
0	0	0	0	0
0	0	0	0	0
0	0	1	1	0
0	0	0	0	0
1	0	0	0	0
0	0	0	0	0
1	0	0	0	0
0	0	0	1	0
0	0	0	0	0
1	0	0	0	1
0	0	1	1	0
1	0	0	1	0
0	0	0	0	0
1	0	0	0	0
1	0	0	0	0
0	0	1	0	0
0	0	1	0	0
6	0	4	4	1
17	17	17	17	17
35.29%	0.00%	23.53%	23.53%	5.88%
custom				

Supplemental Figure 2. Funnel plots of all feature groups.

A. Total sleep time

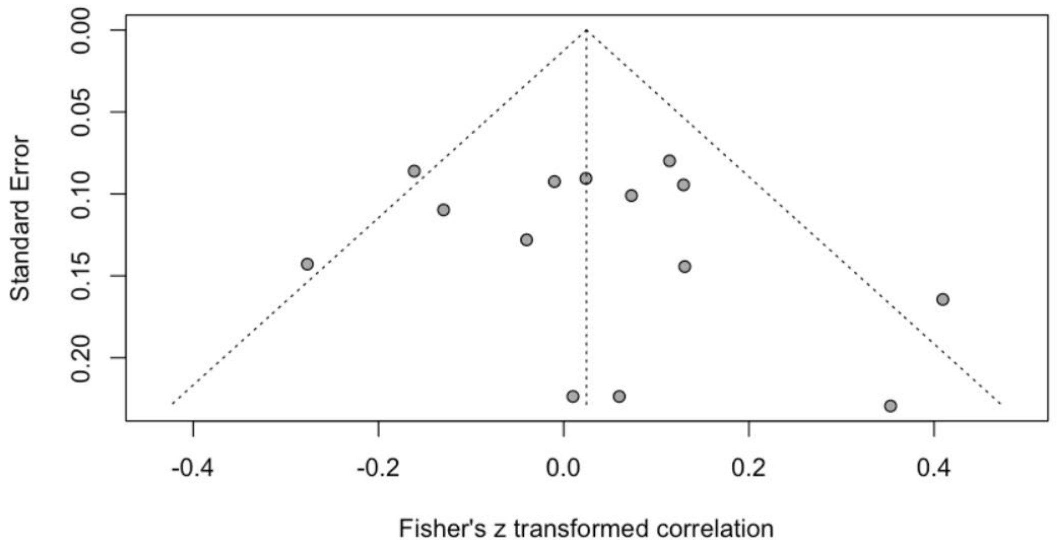

B. Wake after sleep onset

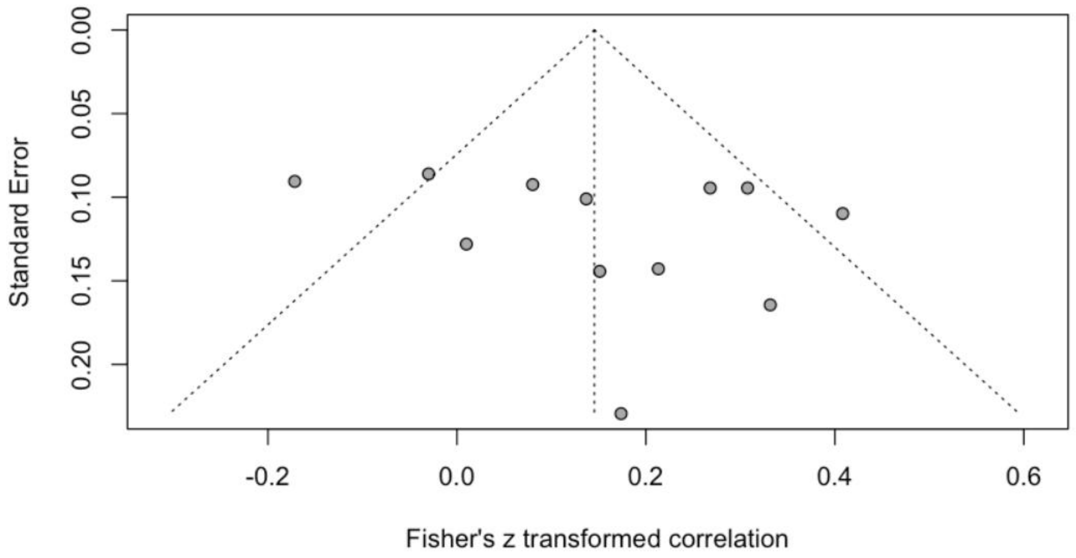

C. Sleep efficacy

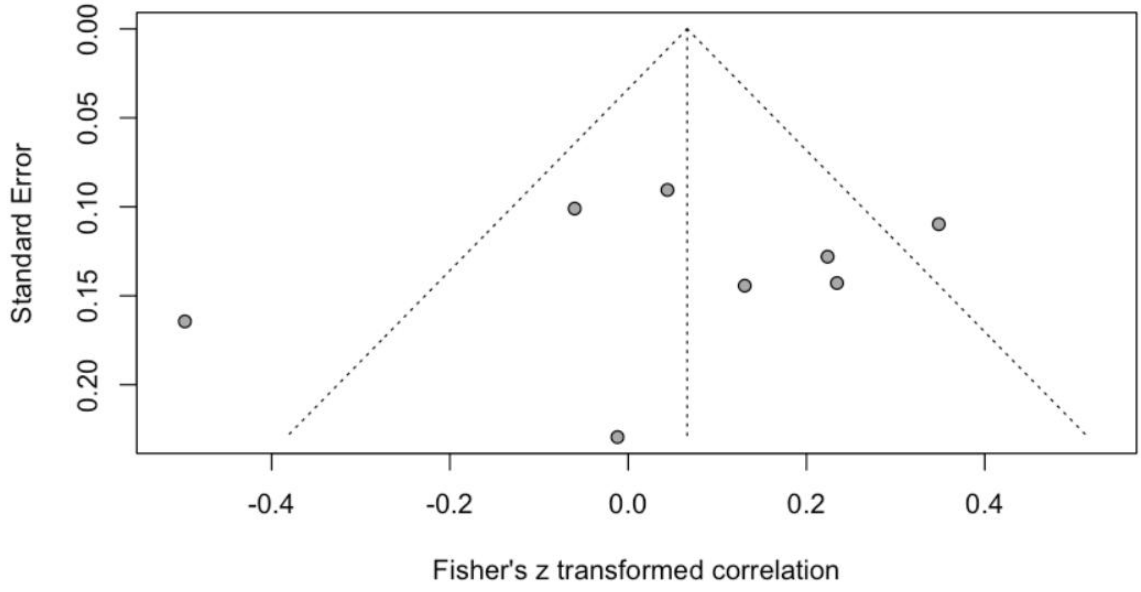

D. Sleep latency

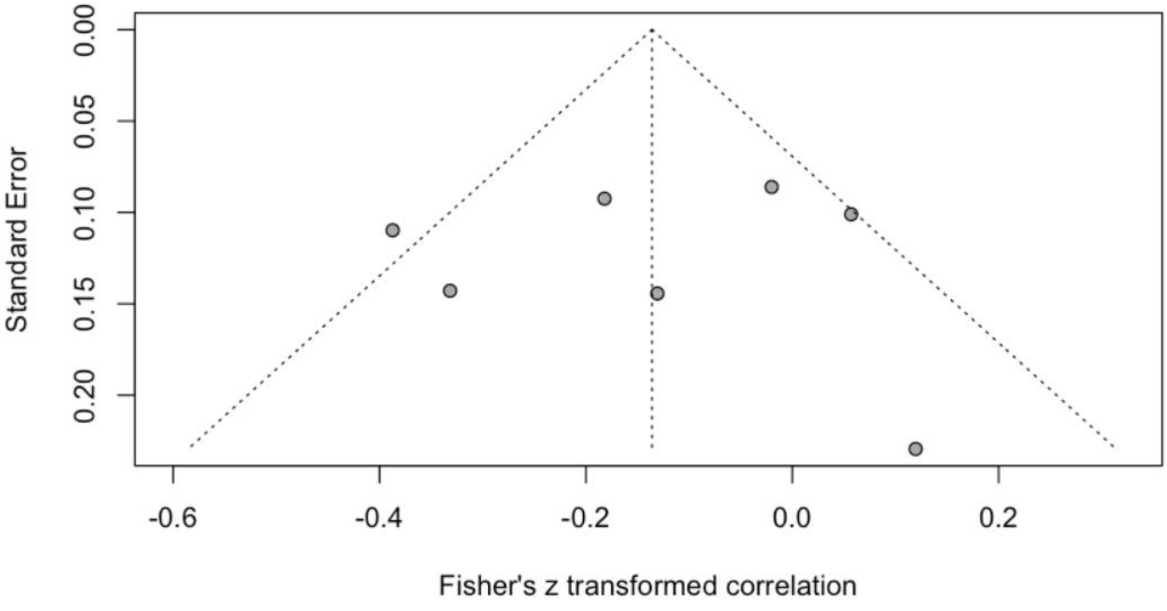

E. Sleep Regularity

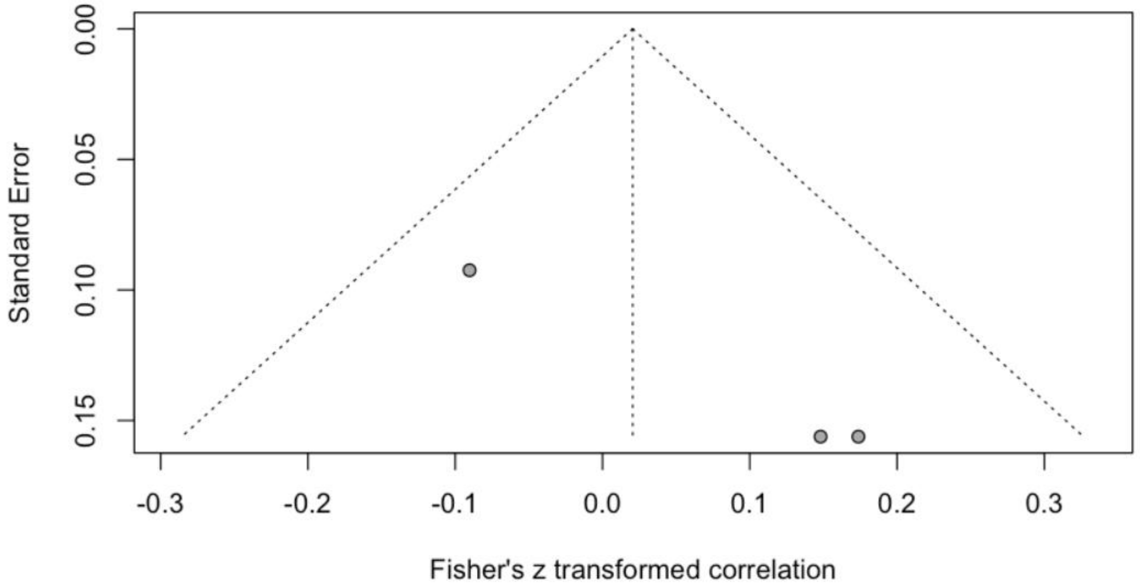

F. Number of awakenings

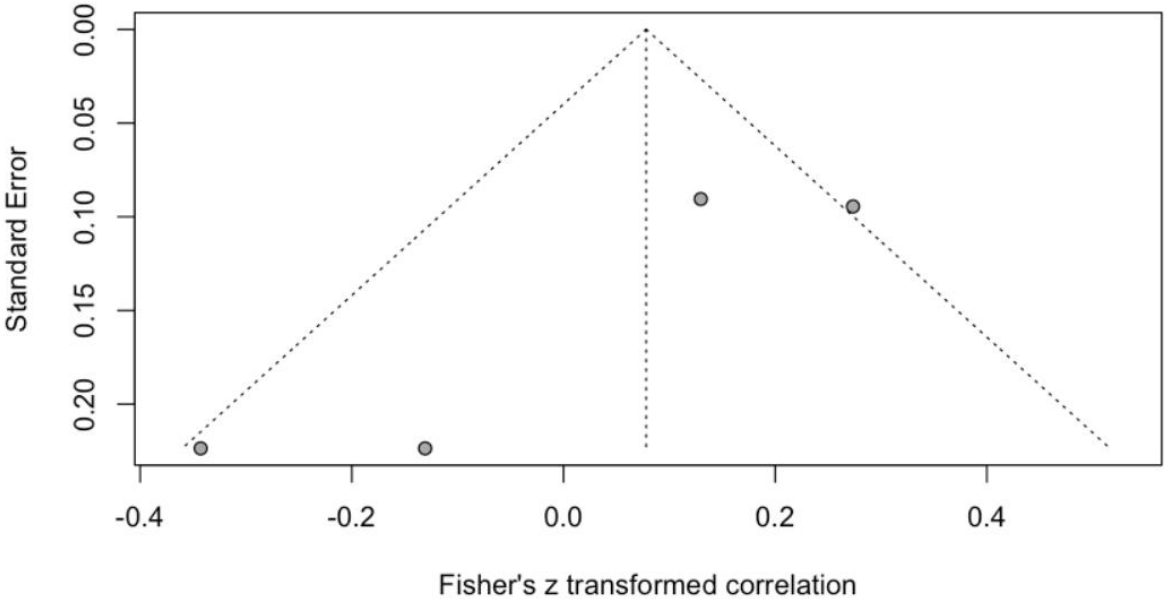

G. Interdaily stability

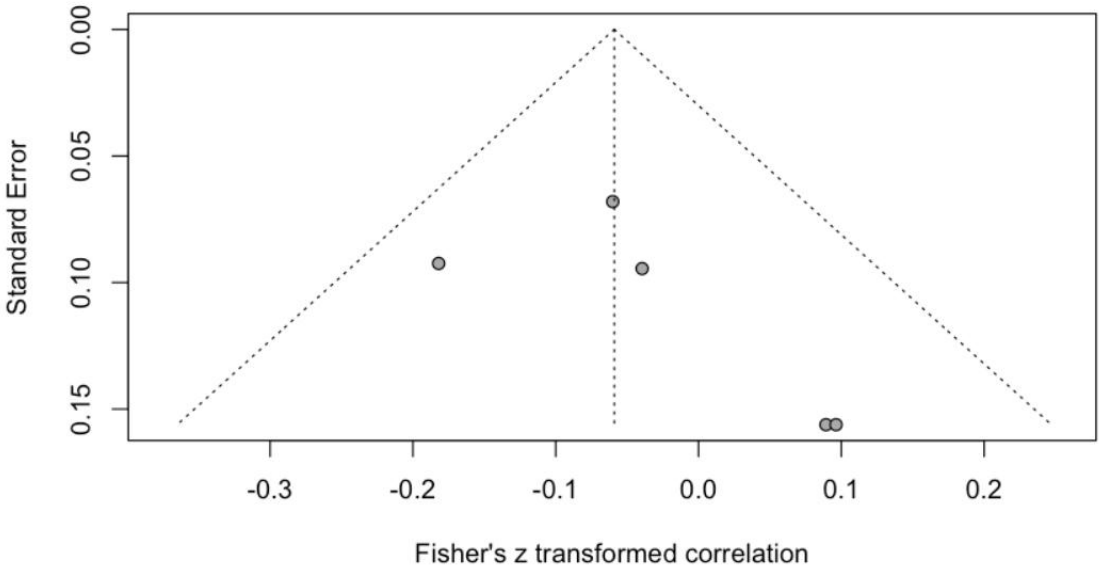

H. Intradaily variability

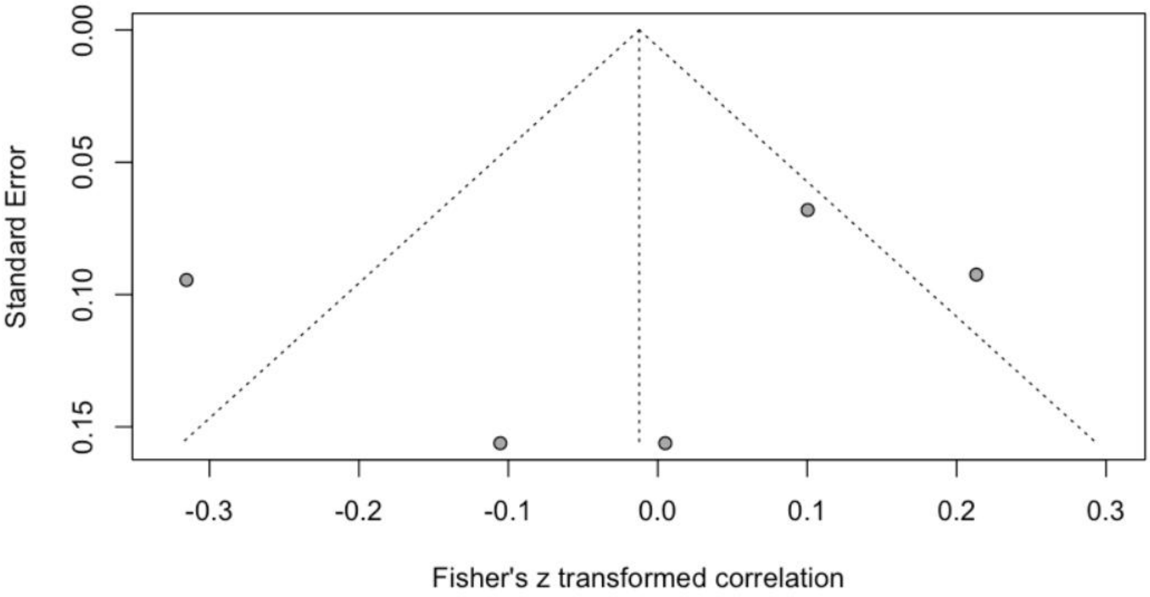

I. Most active 10-h period (M10)

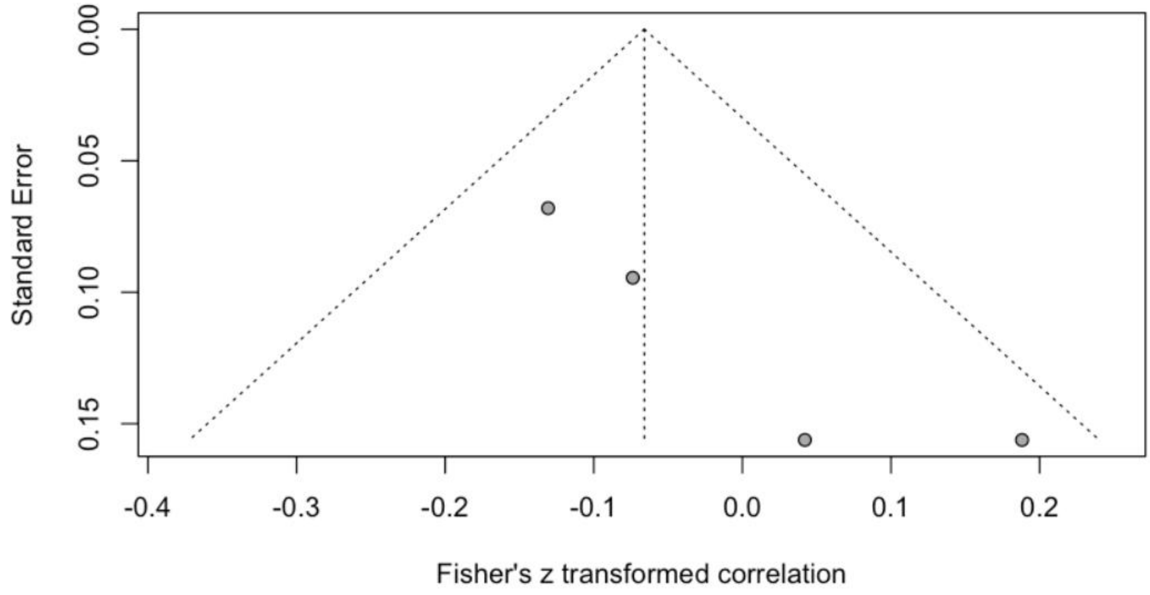

J. Least active 5 hour periods (L5)

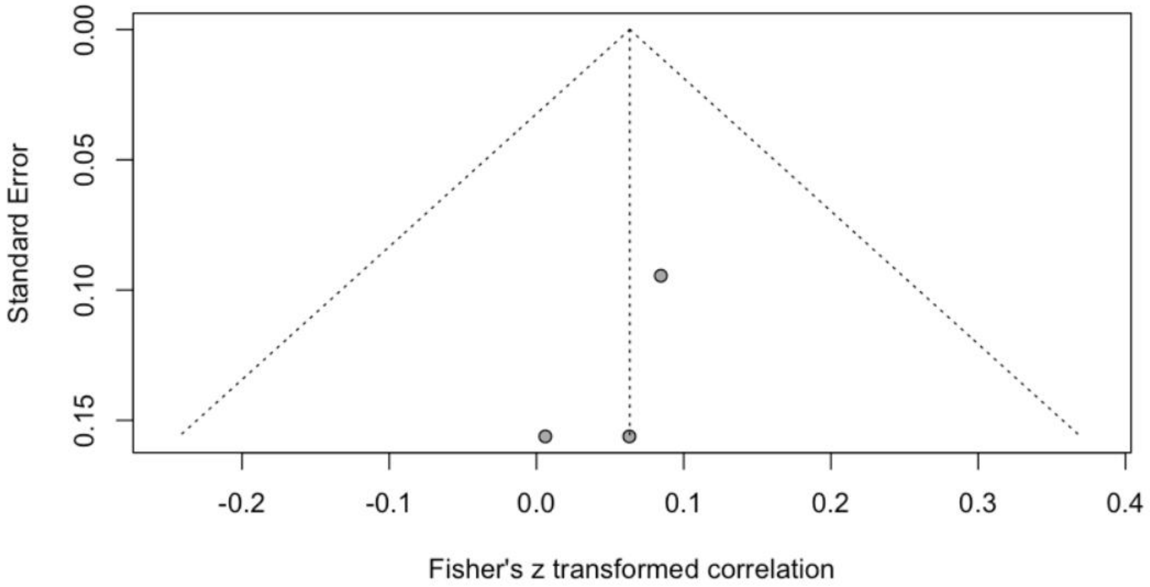

K. Relative amplitude

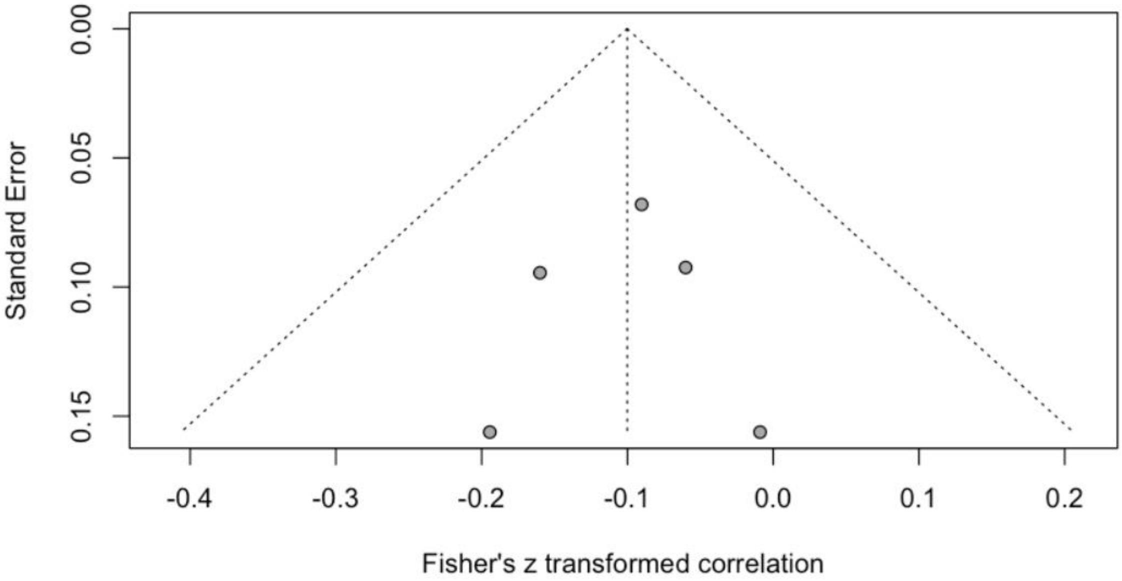

Supplement: Supplementary file 1 — Supplementary information [file 41746_2025_1825_MOESM1_ESM.pdf]
